# Ultralong Nanowires of Cadmium Phosphate Hydroxide Synthesized Using a Cadmium Oleate Precursor Hydrothermal Method and Sulfidation Conversion to Ultralong CdS Nanowires

**DOI:** 10.3390/molecules29020549

**Published:** 2024-01-22

**Authors:** Yu-Qiao Chen, Ying-Jie Zhu, Zhi-Chao Xiong

**Affiliations:** 1 State Key Laboratory of High Performance Ceramics and Superfine Microstructure, Shanghai Institute of Ceramics, Chinese Academy of Sciences, Shanghai 200050, China; chenyuqiao0730@163.com; 2 Center of Materials Science and Optoelectronics Engineering, University of Chinese Academy of Sciences, Beijing 100049, China

**Keywords:** ultralong nanowires, cadmium phosphate hydroxide, cadmium sulfide, oleate precursor, hydrothermal, sulfidation, fire-resistant paper

## Abstract

Ultralong nanowires with ultrahigh aspect ratios exhibit high flexibility, and they are promising for applications in various fields. Herein, a cadmium oleate precursor hydrothermal method is developed for the synthesis of ultralong nanowires of cadmium phosphate hydroxide. In this method, water-soluble cadmium salt is used as the cadmium source, water-soluble phosphate is used as the phosphorus source, and sodium oleate is adopted as a reactant to form cadmium oleate precursor and as a structure-directing agent. By using this method, ultralong nanowires of cadmium phosphate hydroxide are successfully synthesized using CdCl_2_, sodium oleate, and NaH_2_PO_4_ as reactants in an aqueous solution by hydrothermal treatment at 180 °C for 24 h. In addition, a new type of flexible fire-resistant inorganic paper with good electrical insulation performance is fabricated using ultralong nanowires of cadmium phosphate hydroxide. As an example of the extended application of this synthetic method, ultralong nanowires of cadmium phosphate hydroxide can be converted to ultralong CdS nanowires through a convenient sulfidation reaction. In this way, ultralong CdS nanowires are successfully synthesized by simple sulfidation of ultralong nanowires of cadmium phosphate hydroxide under mild conditions. The as-prepared ultralong nanowires of cadmium phosphate hydroxide are promising for applications as the precursors and templates for synthesizing other inorganic ultralong nanowires and have wide applications in various fields.

## 1. Introduction

The apatite group of minerals is a large family of compounds that play important roles in biomedical, industrial, and environmental processes. The apatite-group minerals are hexagonal or pseudohexagonal monoclinic phosphates, arsenates, and vanadates with the general formula M_5_(AO_4_)_3_X, where M = Ba, Ca, Ce, K, Na, Pb, Sr, Y; A = As, P, Si, V; and X = F, Cl, O, OH, H_2_O. Carbonate ions may partially replace the XO_4_ group with the appropriate charge compensation. Materials from the apatite group were studied as host materials for the long-term immobilization of a large number of elements, including cadmium, copper, lead, nickel, uranium, zinc, iodide, and bromide, indicating that members of the apatite group have great potential in environmental remediation techniques [1].

Hydroxyapatite (Ca_10_(PO_4_)_6_(OH)_2_) is one of the most common members of the apatite group, and it is the major inorganic constituent of hard tissues, such as bone and teeth, and has promising applications in various fields [2]. Recently, ultralong hydroxyapatite nanowires were successfully synthesized using the calcium oleate precursor solvothermal/hydrothermal method [3,4]. The as-prepared ultralong hydroxyapatite nanowires exhibit high flexibility and excellent resistance to both high temperature and fire and are excellent building materials for flexible hydroxyapatite-based functional materials with potential applications in various fields [4]. Hydroxyapatite (calcium hydroxyapatite) can host a variety of chemical substituents in its structure. Hydroxyapatite can be substituted with various chemical species. Ca^2+^ ions can be replaced by other metallic ions, such as Mg^2+^, Sr^2+^, Zn^2+^, Pb^2+^, and Ag^+^ ions. For instance, hydroxyapatite-supported Ag_3_PO_4_ nanoparticles with high visible light photocatalytic activity and antibacterial function were synthesized using a cation exchange method [5].

Cadmium phosphate hydroxide (cadmium hydroxyapatite, Cd_5_(PO_4_)_3_(OH), CPH) is a mineral that can be considered a full substitution of Ca^2+^ ions of calcium hydroxyapatite by Cd^2+^ ions. Ca^2+^ ions with a radius of 0.100 nm of calcium hydroxyapatite can be substituted by Cd^2+^ ions with a radius of 0.095 nm, which may result in the contraction of the apatite structure [6]. Hata et al. [7] determined the structure of CPH, which is hexagonal with P6_3_/m, a = 9.335(2) Å, and c = 6.664(3) Å. CPH-based materials have various applications in fields such as catalysis, phosphorescence, and pharmaceutical intermediates.

Different morphologies of CPH were prepared through various synthetic methods. For example, Yasukawa et al. [6] prepared crystallized needle-like CPH structures through a two-step solution method using acetamide. Cd_5_H_2_(PO_4_)_4_·4H_2_O, which is a precursor of CPH, was synthesized from an aqueous solution containing Cd(NO_3_)_2_, (NH_4_)_2_HPO_4_, NH_4_NO_3_, HNO_3_, and acetamide at 100 °C for 1 day during the first step, and then transformed into CPH in aqueous solution containing NH_4_NO_3_ and NH_3_·H_2_O by dissolution and recrystallization at 100 °C for 1–7 days during the second step. Zhu et al. [8] synthesized CPH crystals with various morphologies via a high-temperature mixing method under hydrothermal conditions using solutions of Cd(NO_3_)_2_, (NH_4_)_2_HPO_4_, and ammonia. The pH value had a significant influence on the morphology of the product. The intermediate phase of Cd_5_H_2_(PO_4_)_4_·4H_2_O formed in a weak alkali reaction medium at pH 9, and it took a long time to dissolve and change to bulk-like Cd_5_(PO_4_)_3_(OH) crystals. In a strong alkali reaction medium at pH 11, the intermediate phase of Cd_2_P_2_O_7_·5H_2_O formed and then dissolved and rapidly changed to Cd_5_(PO_4_)_3_(OH) fibers. Cd_3_(OH)_5_NO_3_ crystals formed in the nitrate solution before mixing at 200 °C. Guan et al. [9] synthesized CPH hierarchical structures using a simple template-free hydrothermal method at different temperatures (140~200 °C) for 1~12 h using an aqueous solution containing Cd(CH_3_COO)_2_ and Na_3_PO_4_. By adjusting the initial pH value of the reaction system, various morphologies of Cd_5_(PO_4_)_3_(OH) crystals could be obtained, such as a bunch-like structure consisting of nanosized cuboids, a quasi peanut-like structure consisting of nanoparticles, and a flower-like structure consisting of assembled bundles. They found that pure Cd_5_(PO_4_)_3_(OH) could only absorb UV light with wavelengths shorter than ~350 nm, while hydroxyapatite-supported Ag_3_PO_4_ composites exhibited high photocatalytic activity for the decomposition of methyl orange dye in aqueous solution under visible-light irradiation.

Recently, our research group developed the oleate precursor solvothermal method [3] and synthesized ultralong nanowires of Sr-doped hydroxyapatite and strontium hydroxyapatite (Sr_5_(PO_4_)_3_OH), demonstrating the suitability of the oleate precursor solvothermal method for the synthesis of ultralong nanowires of metal ion-doped hydroxyapatite or other metal hydroxyapatite In this study, for the first time, a cadmium oleate precursor hydrothermal method is developed. Ultralong CPH nanowires are synthesized using this method, and the synthetic conditions are optimized by adjusting reaction parameters. In addition, a flexible inorganic fire-resistant paper is prepared using ultralong CPH nanowires as the building material via a simple vacuum-assisted filtration method. More importantly, ultralong CPH nanowires can be used as the precursor and template and can be converted to ultralong CdS nanowires using a convenient sulfidation method.

## 2. Results and Discussion

**Figure 1:**
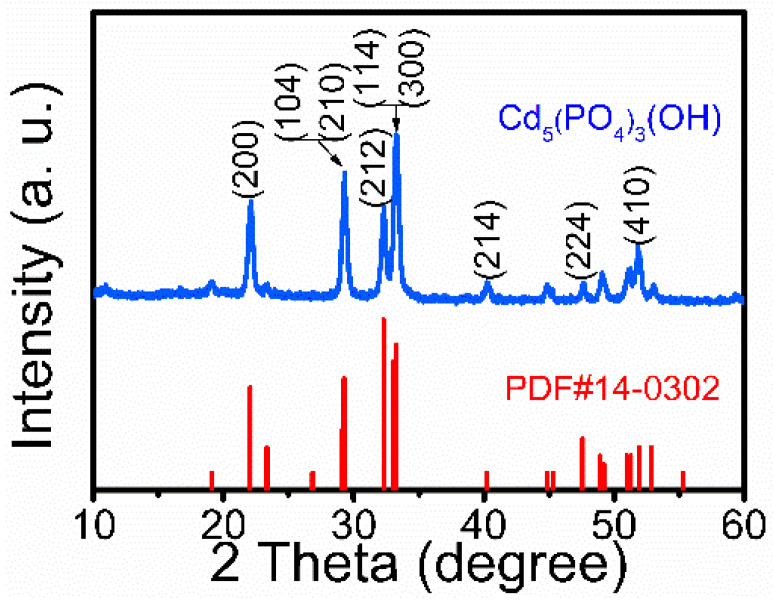
XRD pattern of the as-prepared ultralong CPH nanowires obtained using the cadmium oleate precursor hydrothermal method with an aqueous solution containing CdCl_2_, C_17_H_33_COONa, and NaH_2_PO_4_ at 180 °C for 24 h. The corresponding product is Sample 4 in Table 1.

Ultralong CPH nanowires are synthesized using the cadmium oleate precursor hydrothermal method with an aqueous solution containing CdCl_2_, C_17_H_33_COONa, and NaH_2_PO_4_. The experimental parameters for the preparation of samples are shown in Table 1. Sample 4, consisting of ultralong CPH nanowires synthesized under the optimized conditions, is characterized by XRD, and the obtained XRD pattern is shown in Figure 1. The XRD analysis indicates that the XRD pattern of the product can be well indexed to a single crystal phase of cadmium phosphate hydroxide with a hexagonal structure, which is in good agreement with the standard XRD data (JCPDS No.14-0302).

**Figure 2:**
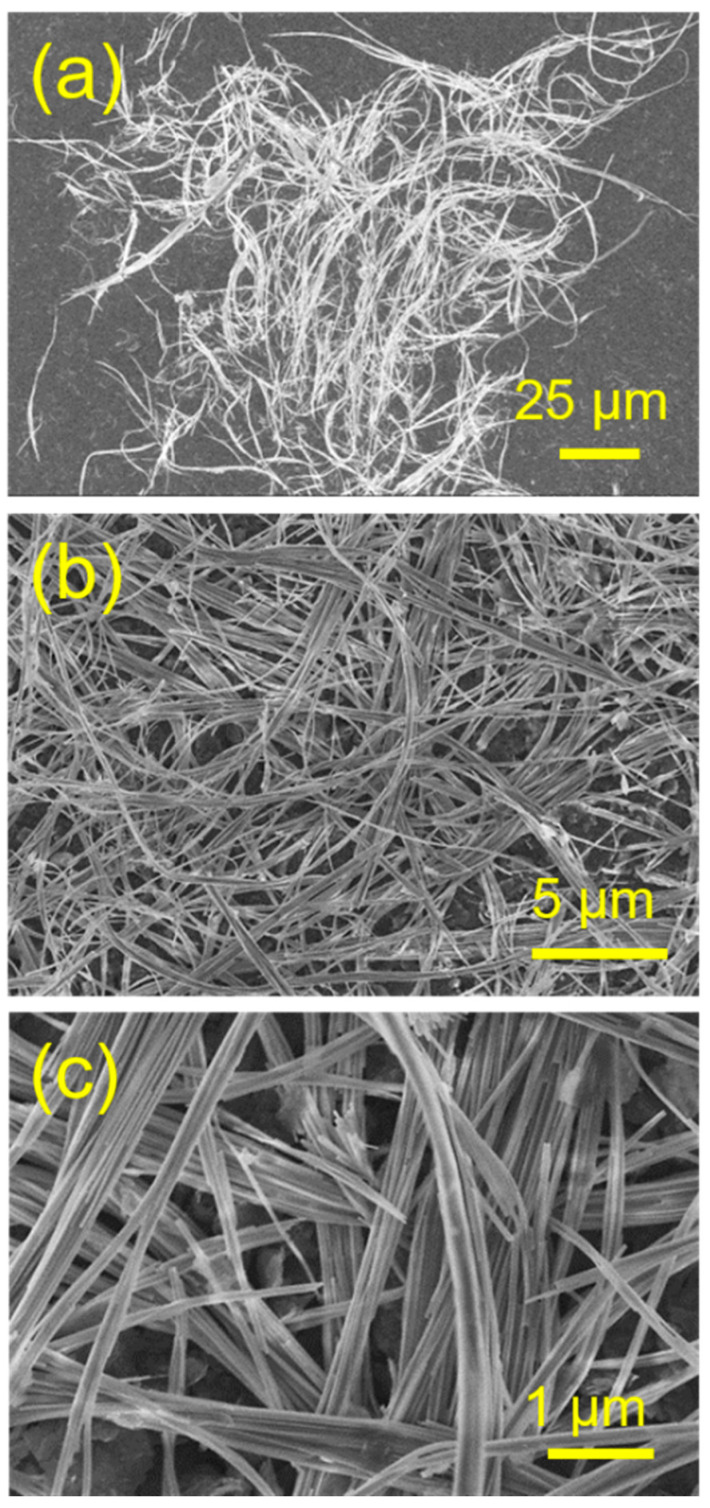
(**a**–**c**) SEM micrographs of the as-prepared ultralong CPH nanowires obtained using the cadmium oleate precursor hydrothermal method with an aqueous solution containing CdCl_2_, C_17_H_33_COONa, and NaH_2_PO_4_ at 180 °C for 24 h. The corresponding product is Sample 4 in Table 1.

Figure 2 shows SEM micrographs of the optimized sample (Sample 4) prepared using the cadmium oleate precursor hydrothermal method with an aqueous solution containing CdCl_2_, C_17_H_33_COONa (2.436 g), and NaH_2_PO_4_ at 180 °C for 24 h. One can see that the product consists of ultralong CPH nanowires with diameters of <100 nm and lengths of several hundred micrometers. The aspect ratios of the as-prepared ultralong CPH nanowires are high (>1000). In addition, in many cases, ultralong CPH nanowires self-assemble along the longitudinal direction to form CPH nanowire bundles with larger diameters. The as-prepared ultralong CPH nanowires are flexible because of their high aspect ratios and ultralong CPH nanowires can bend naturally at any angle. The as-prepared flexible ultralong CPH nanowires can be used as building materials to construct various flexible, functional materials.

**Figure 3:**
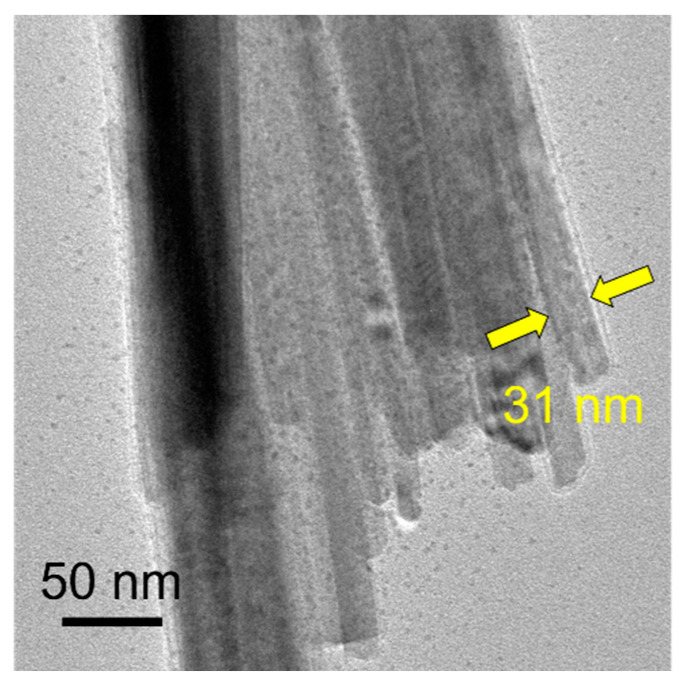
TEM micrograph of the as-prepared ultralong CPH nanowires obtained using the cadmium oleate precursor hydrothermal method with an aqueous solution containing CdCl_2_, C_17_H_33_COONa, and NaH_2_PO_4_ at 180 °C for 24 h. The corresponding product is Sample 4 in Table 1.

**Figure 4:**
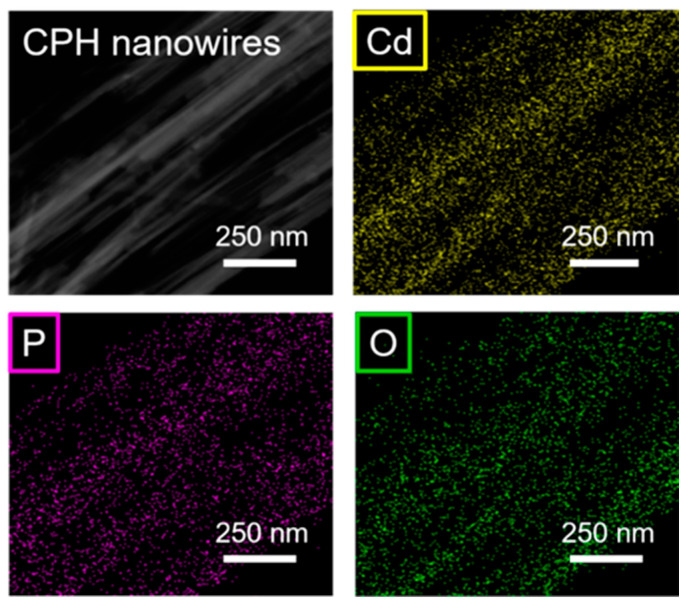
Elemental mapping of Cd, P, and O in the as-prepared ultralong CPH nanowires.

The TEM micrograph in Figure 3 further demonstrates the microstructure of the ultralong CPH nanowires. The diameter of a single CPH nanowire is around tens of nanometers, and the self-assembled nanowire bundles have larger diameters (several hundred nanometers). As shown by the arrows in Figure 3, the diameter of a single ultralong CPH nanowire is about 31 nm. The as-prepared ultralong CPH nanowires are further characterized by elemental mapping, as shown in Figure 4. Elements of Cd, P, and O are detected in the as-prepared ultralong CPH nanowires, and these elements are relatively uniformly distributed in ultralong CPH nanowires.

**Figure 5:**
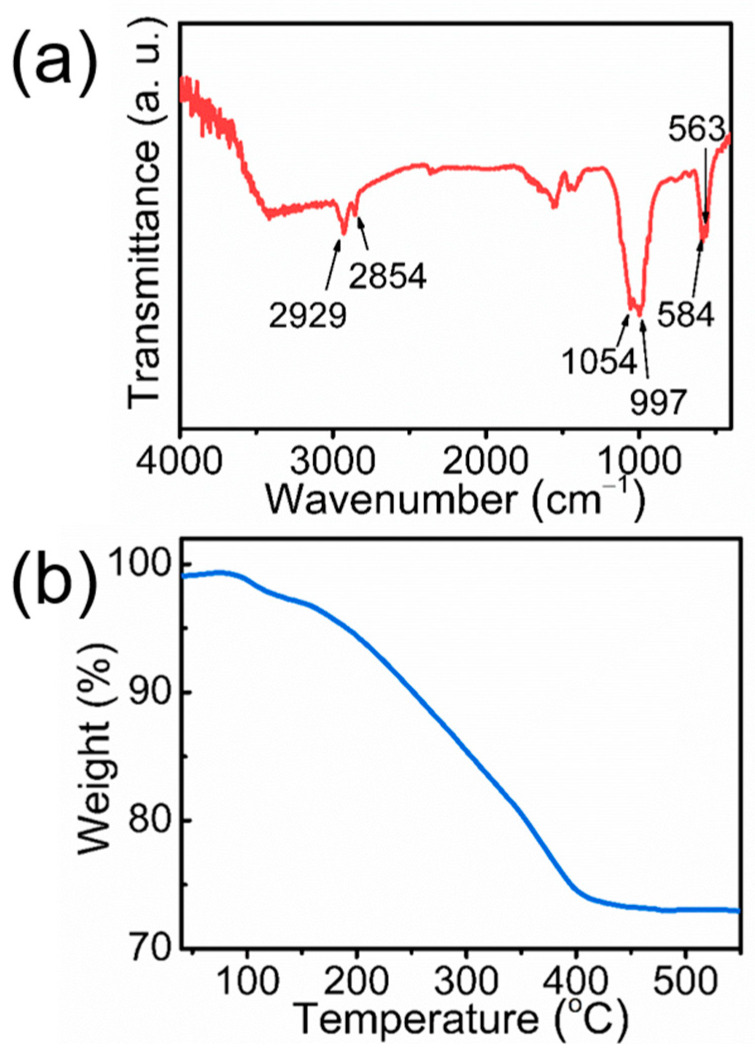
(**a**) Fourier transform infrared (FTIR) spectrum and (**b**) thermogravimetric (TG) curve of the as-prepared ultralong CPH nanowires (Sample 4 in Table 1).

The optimized product of ultralong CPH nanowires in Sample 4 is further characterized using FTIR and TG analysis. The FTIR spectrum (Figure 5a) shows the characteristic absorption peaks and wavenumbers of CPH. The characteristic absorption peaks of the PO_4_^3−^ group in ultralong CPH nanowires are located at 1054, 997, 584, and 563 cm^−1^. The characteristic absorption peaks of the oleate group are located at 2929 and 2854 cm^−1^, indicating that a certain amount of oleate groups adsorbed on the surface of ultralong CPH nanowires has not been completely removed by washing. However, clean ultralong CPH nanowires can be obtained by thorough washing with ethanol and water. As shown by the TG curve in Figure 5b, the weight of the ultralong CPH nanowire sample decreases with increasing temperature. The total weight loss of ultralong CPH nanowires at temperatures of >500 °C is ~27 wt.% due to the loss of adsorbed water and decomposition of adsorbed oleate groups on the surface of ultralong CPH nanowires. The experimental result indicates that the as-prepared ultralong CPH nanowires are composed of approximately 73 wt.% CPH and 27 wt.% adsorbed oleate and water.

**Figure 6:**
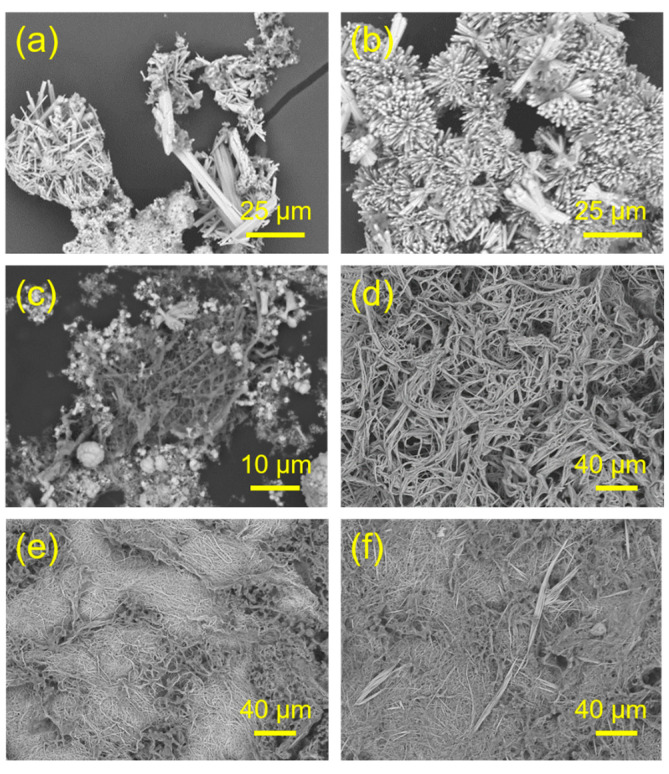
SEM micrographs of the samples prepared using the cadmium oleate precursor hydrothermal method with aqueous solutions containing CdCl_2_, C_17_H_33_COONa, and NaH_2_PO4 and different amounts of sodium oleate at 180 °C for 24 h. (**a**) 0.152 g; (**b**) 0.305 g; (**c**) 1.218 g; (**d**) 2.741 g; (**e**) 3.046 g; (**f**) 3.351 g.

The effect of the added weight of sodium oleate in the reaction system on the morphology of the product is investigated. Figure 6 shows SEM micrographs of the samples prepared using the cadmium oleate precursor hydrothermal method with aqueous solutions containing CdCl_2_, C_17_H_33_COONa, and NaH_2_PO_4_ with different amounts of sodium oleate at 180 °C for 24 h. In the experiments, different weights of sodium oleate ranging from 0.152 g to 3.351 g are used in the reaction system. On the other hand, the added amounts of CdCl_2_·2.5H_2_O and NaH_2_PO_4_·2H_2_O are kept constant. The experimental results reveal that the amount of sodium oleate added to the reaction system has a remarkable effect on the morphology of the resulting product. With the use of a low weight of sodium oleate, the oleate groups react with Cd^2+^ ions to form the cadmium oleate precursor, but there are not enough free oleate groups to act as the structural directing agent for regulation of the growth of CPH nanocrystals along the *c* axis direction. As a result, short CPH nanorods are obtained when a small amount of sodium oleate is used in the reaction system (Figure 6a–c). Moreover, when the weight of sodium oleate is adequate (2.436~3.351 g), the products of ultralong CPH nanowires with high aspect ratios are obtained (Figure 2a–c and Figure 6d–f). The optimal weight of sodium oleate is 2.436 g, and the as-prepared ultralong CPH nanowires are very long (several hundred micrometers) with high flexibility (Figure 2). When the weights of sodium oleate are relatively high (2.741 g~3.351 g), ultralong CPH nanowires with high flexibility can be synthesized, but the lengths and aspect ratios of the ultralong CPH nanowires are smaller than those of the optimized sample (Sample 4). These experimental results reveal that the amount of sodium oleate added has a significant influence on the morphology of the CPH product.

The formation mechanism of ultralong CPH nanowires is described below. During the synthesis of ultralong CPH nanowires using the cadmium oleate precursor hydrothermal method, oleate ions react with Cd^2+^ ions to form the cadmium oleate precursor. During the hydrothermal process, the cadmium oleate precursor reacts with PO_4_^3−^ ions to form the amorphous nuclei, and subsequently, the amorphous nuclei grow into CPH crystals. The oleate groups, as the structural directing agents, are preferentially adsorbed on the a and b crystal planes; therefore, the CPH crystals can grow preferentially along the *c* axis, forming the relatively short CPH nanorods first. Extension of the duration of the hydrothermal process causes the CPH nanorods to preferentially grow along the c axis into ultralong CPH nanowires.

Based on the above discussion, the chemical reactions involved in the formation of ultralong CPH nanowires are as follows:

CdCl_2_ → Cd^2+^ + 2Cl^–^

Cd^2+^ + 2C_17_H_33_COONa → Cd(C_17_H_33_COO)_2_ + 2Na^+^

C_17_H_33_COONa + H_2_O → C_17_H_33_COOH + OH^–^ + Na^+^

NaH_2_PO_4_ → Na^+^ + 2H^+^ + PO_4_^3–^

5Cd(C_17_H_33_COO)_2_ + 3PO_4_^3–^ + OH^–^ → Cd_5_(PO_4_)_3_(OH) + 10C_17_H_33_COO^–^

**Figure 7:**
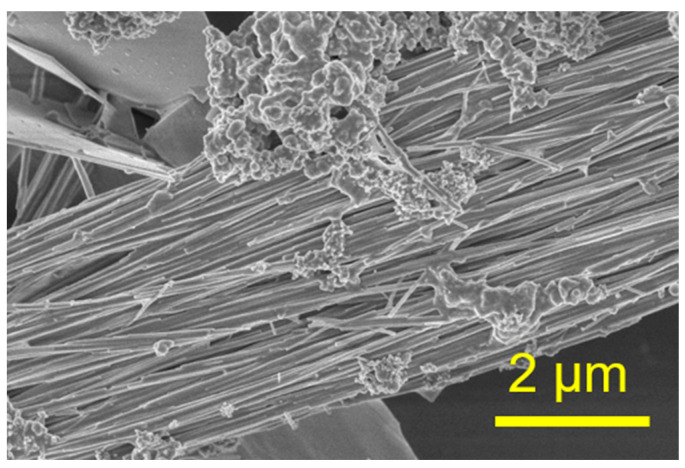
SEM micrograph of ultralong CPH nanowires synthesized using the cadmium oleate precursor hydrothermal method in a 1 L Teflon-lined stainless steel autoclave.

The cadmium oleate precursor hydrothermal method reported herein can also be scaled up for the synthesis of ultralong CPH nanowires. Ultralong CPH nanowires were further synthesized in a 1 L Teflon-lined stainless steel autoclave using the cadmium oleate precursor hydrothermal method. The SEM image of the product obtained using a 1 L synthesis system is shown in Figure 7. The product maintains the morphology of ultralong CPH nanowires. Similar to the product prepared using the small-volume synthesis system, ultralong CPH nanowires obtained using a scaled-up synthesis can self-assemble along the longitudinal direction to form nanowire bundles with larger diameters.

**Figure 8:**
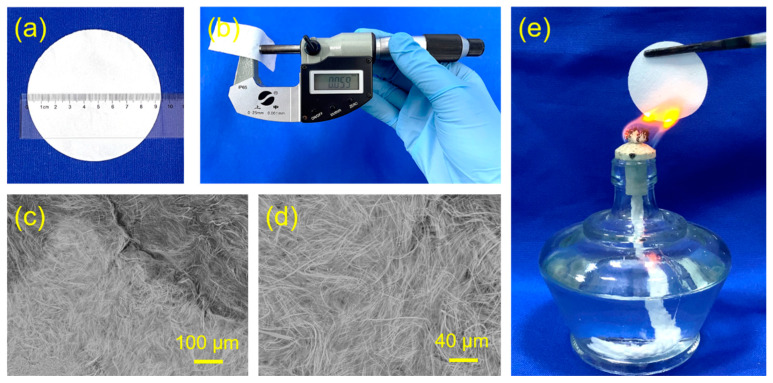
Digital images and surface morphologies (SEM images) of the flexible fire-resistant inorganic paper (CPH nanowire fire-resistant paper) prepared from ultralong CPH nanowires using a vacuum-assisted filtration process. (**a**) A digital image showing a round-shaped flexible CPH nanowire fire-resistant paper with a diameter of 9.5 cm. (**b**) The thickness of the as-prepared flexible CPH nanowire fire-resistant paper is measured to be ~0.059 mm. (**c**,**d**) SEM micrographs showing the surface morphology of the flexible CPH nanowire fire-resistant paper. (**e**) A digital image showing the excellent resistance to both fire and high temperature of the flexible CPH nanowire fire-resistant paper with a diameter of 4.0 cm.

**Figure 9:**
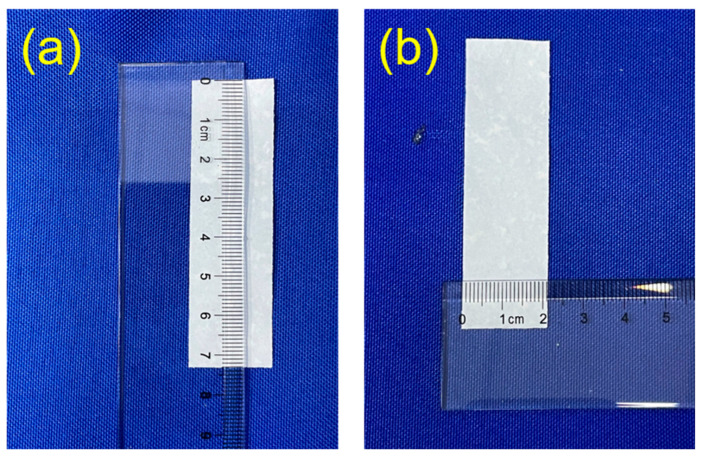
(**a**,**b**) Digital images of the flexible fire-resistant inorganic paper (CPH nanowire fire-resistant paper) prepared using ultralong CPH nanowires through a vacuum-assisted filtration process (a long strip with a size of 73 × 21 mm, which is cut from circular CPH inorganic paper).

Moreover, a new kind of flexible fire-resistant inorganic paper was prepared using ultralong CPH nanowires as the raw material using a vacuum-assisted filtration process. Figure 8a shows a digital image of the as-prepared flexible CPH nanowire fire-resistant paper with a diameter of 9.5 cm. The as-prepared flexible CPH nanowire fire-resistant paper has high flexibility, and it can be bent at any angle. The flexible CPH nanowire fire-resistant paper can be cut into desired shapes, for example, a long strip of 7.3 cm × 2.1 cm × 0.059 mm (Figure 8b and Figure 9). Figure 8c,d show the surface morphology of the as-prepared flexible CPH nanowire fire-resistant paper, and the paper is composed of interwoven and bendable ultralong CPH nanowires with high aspect ratios. Due to its inorganic nature, the as-prepared flexible CPH nanowire fire-resistant paper has outstanding resistance to both high temperatures and flame. As shown in Figure 8e and Video S1 in the Supporting Information, the as-prepared flexible CPH nanowire fire-resistant paper is nonflammable and maintains its integrity without visible damage after being heated in the flame of an alcohol lamp for 1 min. In addition, the as-prepared flexible CPH nanowire fire-resistant paper has a whiteness of ~68.4% without bleaching. As discussed above, a certain amount of oleate groups adsorbed on the surface of ultralong CPH nanowires have not been completely removed during washing, and this will lower the whiteness of the flexible CPH nanowire fire-resistant paper. However, the whiteness of the flexible CPH nanowire fire-resistant paper can be significantly enhanced by using clean ultralong CPH nanowires.

**Figure 10:**
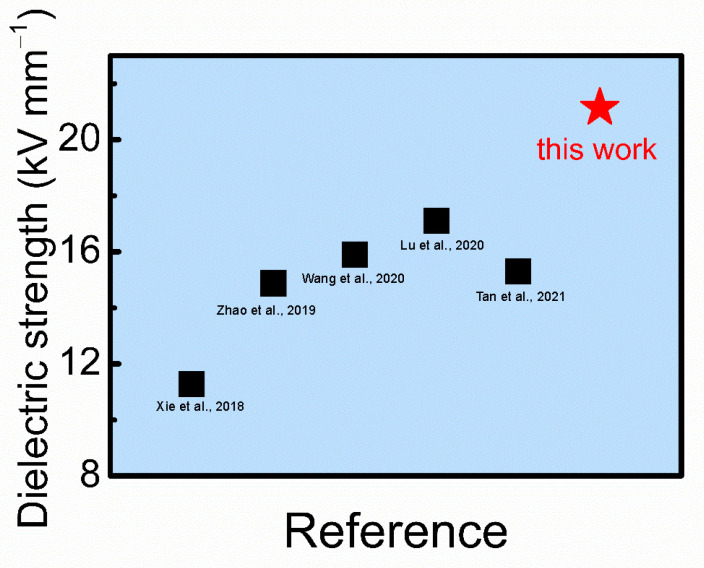
Comparison of dielectric strength between the as-prepared flexible CPH nanowire fire-resistant paper and some electrical insulation materials reported in the literature ([10] Xie et al., 2018; [11] Zhao et al., 2019; [12] Wang et al., 2020; [13] Lu et al., 2020; [14] Tan et al., 2021).

The dielectric breakdown strength is an important property for electrical insulation materials. In this work, we investigated the dielectric breakdown strength of the as-prepared flexible CPH nanowire fire-resistant paper, which is measured to be 21.14 kV mm^−1^. In the as-prepared flexible CPH nanowire fire-resistant paper, ultralong CPH nanowires are interwoven into a networked structure that can uniformly distribute stresses, which can avoid premature electrical breakdown. As shown in Figure 10 and Table 2, the dielectric breakdown strength of the as-prepared flexible CPH nanowire fire-resistant paper is higher than some electrical insulation materials previously reported in the literature.

**Figure 11:**
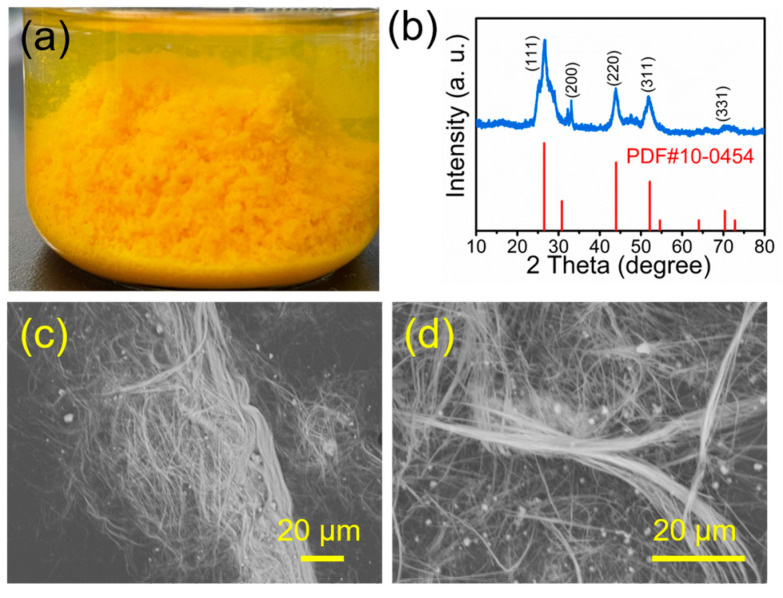
Characterization of ultralong CdS nanowires prepared by sulfidation conversion of ultralong CPH nanowires. (**a**) Digital image, (**b**) XRD pattern, and (**c**,**d**) SEM micrographs of the as-prepared ultralong CdS nanowires.

CdS is a type of valuable group II-VI semiconductor material with superior optoelectronic properties and has a wide range of potential applications in the fields of biosensors [15], photocatalysis [16], solar cells [17], and light-emitting diodes [18]. In this work, we developed a convenient sulfidation conversion method for the synthesis of ultralong CdS nanowires using ultralong CPH nanowires as both the precursor and template. By using this strategy, ultralong CdS nanowires have been successfully synthesized (Figure 11). As shown in Figure 11b, the product of ultralong CdS nanowires is characterized by XRD, which is in good agreement with the standard data (JCPDF No.10-0454), indicating the successful formation of ultralong CdS nanowires with cubic crystal structure. In comparison to other methods of CdS material synthesis, the preparation method reported in this study for flexible ultralong CdS nanowires is facile, environmentally friendly, and low-cost.

**Figure 12:**
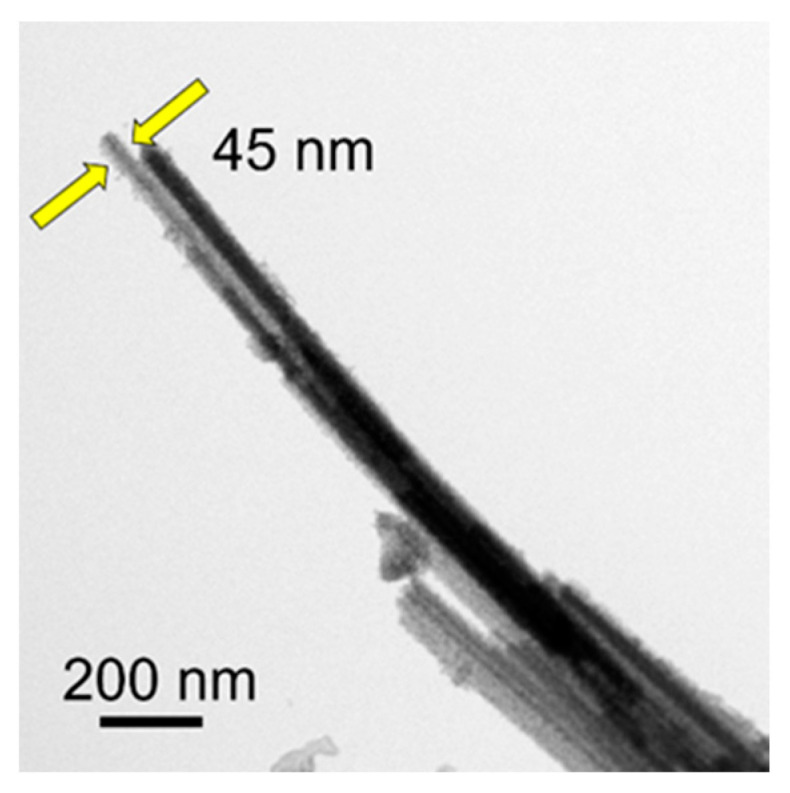
TEM micrograph of the as-prepared ultralong CdS nanowires.

The SEM micrographs in Figure 11c,d show that the product maintains the nanowire morphology with high aspect ratios and high flexibility. In addition, the microstructure of ultralong CdS nanowires is shown in the TEM micrograph in Figure 12. Similar to ultralong CPH nanowires, the diameters of single ultralong CdS nanowires are tens of nanometers, and ultralong CdS nanowires self-assemble along their longitudinal direction to form nanowire bundles with larger diameters. As shown by the arrows in Figure 12, the diameter of a single ultralong CdS nanowire is 45 nm.

The chemical reactions involved in the conversion of ultralong CPH nanowires to ultralong CdS nanowires are as follows:

Na_2_S·9H_2_O → 2Na^+^ + S^2–^ + 9H_2_O

Cd_5_(PO_4_)_3_(OH) + 5S^2–^ → 5CdS + 3PO_4_^3–^ + OH^–^

**Figure 13:**
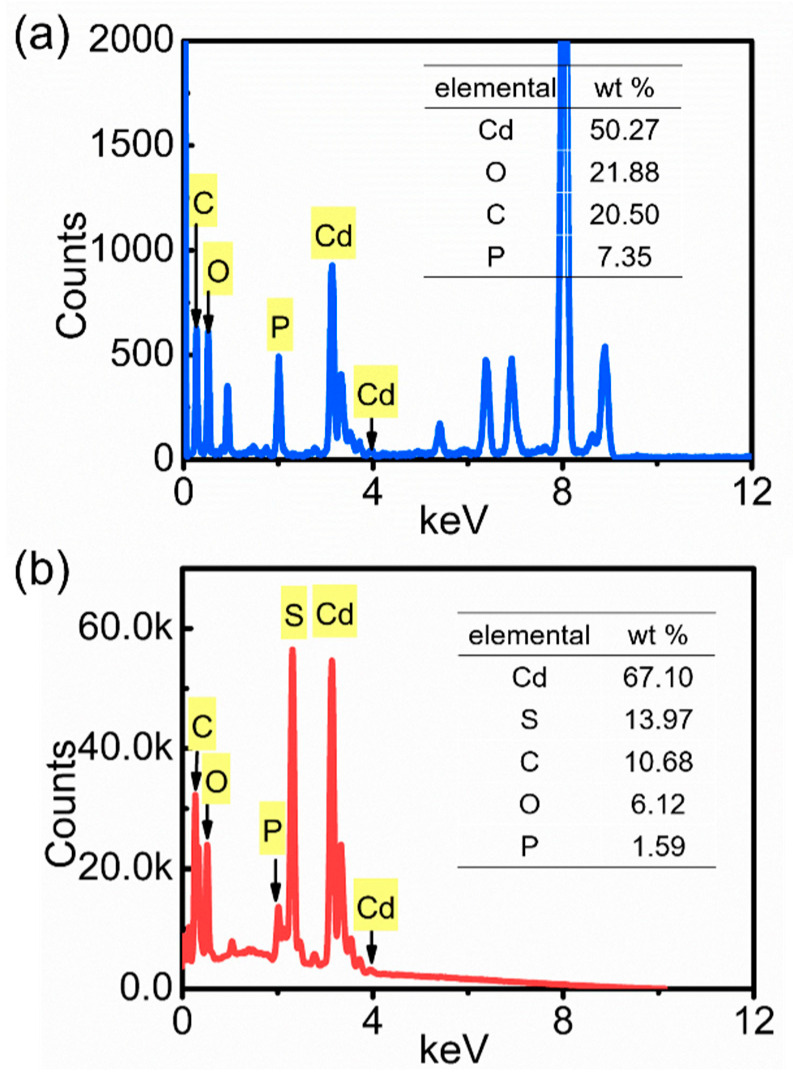
EDX spectra of the as-prepared ultralong CPH nanowires (**a**) and ultralong CdS nanowires (**b**).

**Figure 14:**
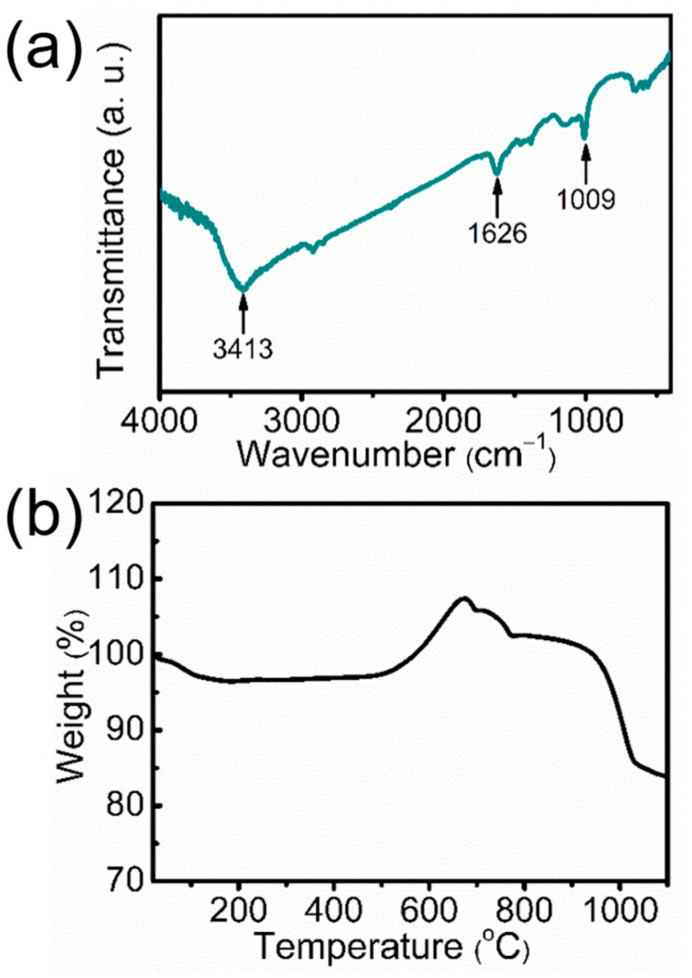
(**a**) FTIR spectrum and (**b**) TG curve of the as-prepared ultralong CdS nanowires.

The energy dispersive X-ray spectroscopy (EDX) spectra of ultralong CPH nanowires and ultralong CdS nanowires are shown in Figure 13. A significant decrease in the P element and the appearance of the S element can be seen in the EDX spectrum of the ultralong CdS nanowires, confirming the conversion of CPH to CdS. The FTIR spectrum of the as-prepared ultralong CdS nanowires (Figure 14) also confirms the formation of CdS. There are two characteristic peaks at 1626 and 1009 cm^−1^, which are attributed to the binding vibration of C–O bonds and the vibration of Cd–S bonds in CdS, respectively, implying that a small amount of oleate groups are adsorbed on the surface of the ultralong CdS nanowires. The peak at 3413 cm^−1^ is attributed to the stretching vibration of –OH, indicating the presence of a small amount of adsorbed water on the surface of ultralong CdS nanowires.

As shown by the TG curve in Figure 14b, the weight loss during the early stage (~3 wt.%) in an air atmosphere from 25 °C to 440 °C corresponds to the water and oleate groups adsorbed on the surface of ultralong CdS nanowires. The weight gain in the TG curve corresponds to the oxidation of CdS to form CdSO_4_ at temperatures higher than ~450 °C in air and continues to be oxidized to ultimately form solid CdO and SO_2_ gas, which correspond to the subsequent weight loss.

## 3. Experimental Section

### 3.1. Materials

Sodium oleate (C_17_H_33_COONa), cadmium chloride hemi(pentahydrate) (CdCl_2_·2.5H_2_O), and sodium dihydrogen phosphate dihydrate (NaH_2_PO_4_·2H_2_O) were purchased from Sinopharm Chemical Reagent Co., Ltd. (Shanghai, China). Ethanol was obtained from Shanghai Lingfeng Chemical Reagent Co., Ltd. (Shanghai, China). Sodium sulfide nonahydrate (Na_2_S·9H_2_O) was purchased from Adamas Reagent Co., Ltd. (Shanghai, China). All chemicals were of analytical grade and used as received without further purification.

### 3.2. Synthesis of Ultralong CPH Nanowires

The typical experimental procedure for the synthesis of ultralong CPH nanowires using the cadmium oleate precursor hydrothermal method is described below. First, 2.436 g of C_17_H_33_COONa, 0.475 g of CdCl_2_·2.5H_2_O, and 0.281 g of NaH_2_PO_4_·2H_2_O were completely dissolved in 25 mL of deionized water to form solutions A, B, and C, respectively, under magnetic stirring. Then, solutions B and C were successively added to solution A under magnetic stirring at 20 min intervals, and the resultant reaction system was further magnetically stirred for 10 min. The obtained reaction mixture was transferred into a 100 mL Teflon-lined stainless-steel autoclave, sealed, heated at 180 °C for 24 h, and naturally cooled to room temperature. The resulting product was collected by centrifugation and washed with ethanol and deionized water three times, respectively. Finally, ultralong CPH nanowires were obtained. The CPH nanowire inorganic paper was obtained by filtering an aqueous suspension containing ultralong CPH nanowires through a hydrophilic mixed cellulose esters membrane filter paper with a diameter of 47 mm.

### 3.3. Preparation of Ultralong CdS Nanowires

First, 1.000 g of ultralong CPH nanowires and 16.152 g of Na_2_S·9H_2_O were uniformly dispersed/dissolved in 200 mL of deionized water, then the mixture was magnetically stirred at 80 °C for 6 h. The obtained product was collected and thoroughly washed with deionized water to obtain ultralong CdS nanowires.

### 3.4. Characterization

Scanning electron microscopy (SEM) micrographs were obtained using a field-emission scanning electron microscope (SEM, Hitachi S-4800; Hitachi TM-3000, Tokyo, Japan). Transmission electron microscopy (TEM) micrographs were obtained using a field-emission transmission electron microscope (JEM-2100F, JEOL, Tokyo, Japan). X-ray powder diffraction (XRD) patterns were obtained using an X-ray diffractometer (Rigaku D/max 2550 V, Cu Kα radiation, λ = 1.54178 Å). Fourier transform infrared (FTIR) spectroscopy was measured using an FTIR-7600 spectrometer (Lambda Scientific, Edwardstown, Australia). The thermogravimetric (TG) analysis was carried out using a simultaneous thermal analyzer (STA 409/PC, Netzsch, Bavaria, Germany) at a heating rate of 10 °C min^−1^ in flowing air. The dielectric breakdown strength was measured using a voltage tester (YD 2665, Changzhou Yangzi Electronics Co., Ltd., Changzhou, China) under gradually increasing voltage in air.

## 4. Conclusions

In summary, we developed a cadmium oleate precursor hydrothermal method for the synthesis of ultralong CPH nanowires using cadmium oleate as a precursor. In this method, water-soluble cadmium salt is used as the cadmium source, water-soluble phosphate is used as the phosphorus source, sodium oleate is adopted as a reactant to form cadmium oleate precursor and as a structure-directing agent, and water is used as the only solvent without any organic solvent. The as-prepared ultralong CPH nanowires can be further constructed into highly flexible, fire-resistant inorganic paper with excellent nonflammability and high-temperature resistance using a simple vacuum-assisted filtration method. The added amount of sodium oleate in the reaction system has a significant effect on the morphology of the product, and the experimental conditions for synthesizing ultralong CPH nanowires are optimized. Furthermore, ultralong CPH nanowires as the precursor and template can be successfully transformed into ultralong CdS nanowires using sulfidation treatment under mild conditions. This study provides a promising method for the convenient and scalable preparation of ultralong CPH nanowires, fire-resistant inorganic paper, and ultralong CdS nanowires, which is expected to be applied in various fields including electrical insulation, fire retardance, photocatalysis, chemical pigments, photoresistors, photodiodes, and solar cells. In addition, the as-prepared ultralong CPH nanowires are promising for applications as the precursor and template for synthesizing other inorganic ultralong nanowires and have wide applications in various fields.

## Figures and Tables

**Table 1:** Experimental parameters for the synthesis of the samples using the cadmium oleate precursor hydrothermal method with aqueous solutions containing CdCl_2_, C_17_H_33_COONa, and NaH_2_PO_4_ at 180 °C for 24 h.

Sample	C_17_H_33_CO_2_Na	CdCl_2_·2.5H_2_O	NaH_2_PO_4_·2H_2_O	Water	SEM Image
1	0.152 g	0.475 g	0.281 g	75 mL	Figure 6a
2	0.305 g	0.475 g	0.281 g	75 mL	Figure 6b
3	1.218 g	0.475 g	0.281 g	75 mL	Figure 6c
4	2.436 g	0.475 g	0.281 g	75 mL	Figure 2a–c
5	2.741 g	0.475 g	0.281 g	75 mL	Figure 6d
6	3.046 g	0.475 g	0.281 g	75 mL	Figure 6e
7	3.351 g	0.475 g	0.281 g	75 mL	Figure 6f

**Table 2:** Comparison of dielectric strength between the as-prepared flexible CPH nanowire fire-resistant paper and some electrical insulation materials reported in the literature.

Materials	Dielectric Strength (kV mm^−1^)	Reference
SiO_2_ nanoparticles and polyimide fibers	11.27	[10]
Aramid fiber and mica-nanofibrillated cellulose	14.87	[11]
Polytetrafluoroethylene and TiO_2_	15.90	[12]
Mica and nanofibrillated cellulose	17.10	[13]
meta-aramid paper	15.30	[14]
Ultralong CPH nanowires	21.14	This study

## Data Availability

Data is contained within the article/Supplementary Materials, further inquiries can be directed to the corresponding author/s.

## Supplementary Materials

The following supporting information can be downloaded at: https://www.mdpi.com/article/10.3390/molecules29020549/s1. Video S1: Video of the ultralong CPH nanowire-based fire-resistant paper, which is nonflammable and high-temperature-resistant in the alcohol lamp flame.
